# Vaginismus

**Published:** 1868-10

**Authors:** N. S. Davis

**Affiliations:** Chicago


### Vaginismus.

BY PROF. N. S. DAVIS, M. D., OF CHICAGO.

Mrs. H., with her husband, from a distant part of the State, consulted me on the 17th of March, 1868. She stated that she had been married over three years, and, though enjoying fair general health, and feeling the usual sexual desires, had never been able to allow sexual intercourse with her husband, on account of the *terrible* suffering that every attempt had produced.

On attempting to make a vaginal examination with the index finger, it was found necessary to bring her fully under the influence of chloroform before it could be completed. The sphincter at the entrance of the vagina contracted as closely around the finger as the sphincter ani generally does; and without the chloroform, she shrank with terror as the finger separated the labia. Finding no malformation of the parts beyond the entrance of the vagina, except an apparent shortness of the vagina and smallness of the neck of the uterus, she was advised to undergo an operation, such as has been recommended by Dr. J. Marion Sims. This, she and her husband readily assented to; and, on the following day (March 18th,) having had the bowels freely moved, so that the rectum should be empty, I proceeded with the operation, assisted by Dr. S. A. McWilliams and two students. The patient was brought into a state of complete anæsthesia by the cautious use of chloroform, and then placed in the same position on the table as for the performance of lithotomy. The labia were separated by the assistants and a portion of the mucus and submucus tissue, corresponding with the original attachments of the hymen, was dissected away with the scissors and forceps, and two incisions were made in the posterior part of the vagina; each commencing about two inches up, one to the right and the other to the left, making them converge into one on the central line posteriorly, and then extending as one incision outward to the edge of the perineum. The incisions, when completed, resembled in shape the letter Y, and were deep enough to sever the dense, sub-mucus cellular tissue, and the fibres of the sphincter vagina muscle. The operation was completed by placing a soft sponge in the vagina and removing the patient into her bed. The hemorrhage was inconsiderable, and under the influence of a moderate dose of morphine, the patient rested comfortably for several hours. The next morning the sponge was removed, the vagina washed out with water, a small-sized vaginal dilator inserted, and allowed to remain five minutes. From this time, the dilator was inserted every day during the first week, allowing it to remain from fifteen to thirty minutes. During the second week, one of a larger size was used in the same manner. During the third week, the dilator was introduced only every second day, one of medium size entering the vagina with but little pain or resistance.

On the 15th of April, the treatment was discontinued and the patient permitted to return home. The incisions had entirely healed, and a letter, received from her husband soon after, announced that all serious obstruction to sexual intercourse had been removed. The lady was again in the city, a few days since (August 21st,) and assured me that the operation had been entirely successful.—*Chicago Medical Examiner.*

